# Family tree database of the National Health Information Database in Korea

**DOI:** 10.4178/epih.e2019040

**Published:** 2019-10-01

**Authors:** Yeon-Yong Kim, Hae-young Hong, Kyu-Dong Cho, Jong Heon Park

**Affiliations:** 1 Department of Big Data, National Health Insurance Service, Wonju, Korea; 2 Department of Economics, University of Wisconsin, Madison, WI, USA; 3 Department of Benefits Strategy, National Health Insurance Service, Wonju, Korea

**Keywords:** Family, Database, Family relations, Interpersonal relations

## Abstract

We constructed the family tree database (DB) by using a new family code system that can logically express interpersonal family relationships and by comparing and complementing health insurance eligibility data and resident register data of the National Health Information Database (NHID). In the family tree DB, Parents and grandparents are matched for more than 95% of those who were born between 2010 and 2017. Codes for inverse relationships and extended relationships are generated using sequences of the three-digit basic family codes. The family tree DB contains variables such as sex, birth year, family relations, and degree of kinship (maximum of 4) between subjects and family members. Using the family tree DB, we find that prevalence rates of hypertension, diabetes, ischemic heart disease, cerebrovascular disease, and cancer are higher for those with family history. The family tree DB may omit some relationships due to incomplete past data, and some family relations cannot be uniquely determined because the source data only contain relationships between head and members of the household. The family tree DB is a part of the NHID, and researchers can submit requests for data on the website at http://nhiss.nhis.or.kr. Requested data will be provided after approval from the data service review board. However, the family tree DB can be limitedly provided for studies with high public value in order to maximize personal information protection.

## INTRODUCTION

The National Health Insurance (NHI) is a social health insurance system in Korea, which achieved universal coverage of all Korean residents in 1989. Also, all healthcare providers are mandatorily enrolled in the NHI. The National Health Insurance Service (NHIS) is a single insurer, and fee-for-service is the main payment system [1].

The NHIS has demographic information (birth, death, residence, household composition, etc.), socioeconomic information (employer, income, wealth, etc.), disability registration information, in order to manage the eligibility of all residents and impose insurance contributions. Also, the NHIS has a wide range of health risk factors, as it operates the national health screening programs such as infant/adult general check-ups, cancer screening, etc. In addition, it also has detailed information on health care utilization (medical procedures, medications, therapeutic materials) submitted by medical providers for reimbursement. To meet the demands for the data for various research purposes, the NHIS created the National Health Information Database (NHID) in 2012 as a research database (DB), excluding identification information such as resident registration number [2]. In 2018, to further meet the demand for variables such as socio-demographic information, the NHID was redesigned to consist of demographic, geographic, sociological and economic information, social resources, health behaviors, and utilization of health care.

Family relation is known as a relevant factor in the biological aspects of disease and family history [3]. Recently, it receives attention as an important socio-structural factor associated with low birth rate, aging, health issues related to family structure, such as the elderly living alone and single-person households [4]. Furthermore, it is reported that family relation affects mental health as it relates to social trust and social networks [5]. Not only in academic research but also in policy research, accurate information on family relations is essential to make effective health policies and social policies.

However, there have been few source data that provide accurate family relationships. Although there are some survey data having information on family relations, most of them only include relationships to the heads of household, and thus, it is hard to determine the relationships between other household members. The health insurance eligibility data and resident registration data owned by NHIS also have similar limitations, having only codes for relationships to heads of household. To overcome such limitations, we develop a code system to logically express interpersonal relationships within families and establish a DB of interpersonal family relationships of the entire population.

## DATA RESOURCE AREA AND POPULATION COVERAGE

The family tree DB is created by comparing and complementing health insurance eligibility data and resident register data in the NHID to obtain more accurate family relationships. The family tree DB basically covers the same population as the NHID, but it should be noted that the health insurance eligibility data and the resident register data are missing some data before 2002 and 2004, respectively, because of incomplete electronization. The family tree DB does not have a time variable like reference year; instead, it only shows the relationship between two individuals as long as they have ever had a kinship relationship. For example, the family tree DB only shows the relationship that person A is person B’s parent. Therefore, when examining matching rates of the family tree DB, we restrict ourselves to only those who lived in Korea in 2017 to set the denominators. Table 1 shows the parent-child and grandparent-child matching rates of the family tree DB by birth year of child for those living in Korea in 2017. Among the population born in the 2010s, 99.6% are matched with a parent or a grandparent; however, among the population born before the 1950s, 34.3% of men and 5.7% of women are matched with a parent or a grandparent.

Table 2 shows the total number of matched relatives by degree of kinship and by type of kinship for those living in Korea in 2017. The family tree DB stores relationships up to the fourth degree of kinship. Men and women born in the 1950s or before have the largest number of first-degree kins matched. Men born in the 1970s and women born in the 1950s or before have the largest number of second-degree kins matched. Men and women born in the 1970s have the largest number of third-degree kins matched. Men and women born in the 1990s have the largest number of fourth-degree kins matched. Also, men born in the 1970s and women born in the 1950s or before have the largest number of both consanguineous and affinal kins matched. This suggests that the numbers of relatives matched are greater for the elderly.

## MEASURES

### Family code and variables

**Figure 1.**
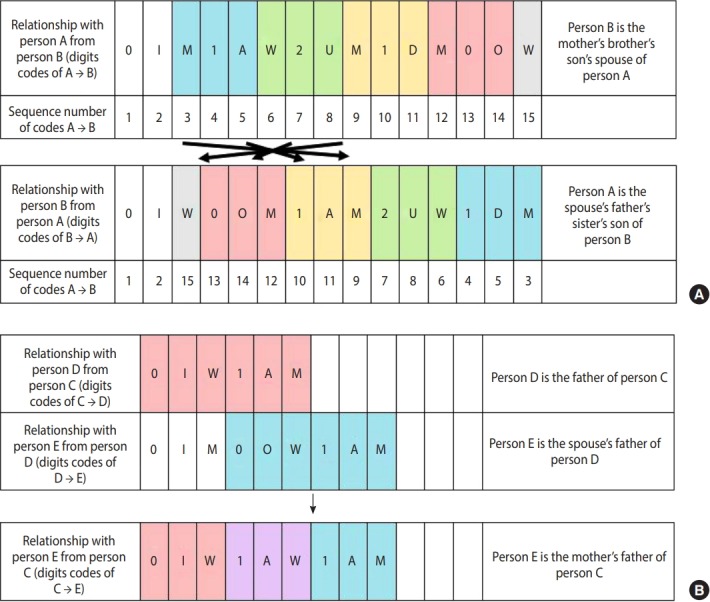
Examples of family codes for the (A) inverse and (B) extended relationship. Three-digit basic family codes (ex. 0IM, 1AW) are specified in Table 3.

We develop a new family code system to identify family relationships between individuals from the limited information on relationships to head of household. The family code is a sequence of three-digit basic codes that are required to connect an individual to their kin. The first digit of a three-digit basic code indicates the degree of kinship, which can take values 0, 1, or 2. The second digit is a character that differentiates self, spouse, parent, child, older sibling, and younger sibling among the same degree of kinship. The third digit indicates gender, which takes values M, W, or X (Table 3). In other words, three-digit basic codes are only assigned to the following relationships which cannot be decomposed into other relationships: self, spouse, parent, child, and sibling for both genders. All family codes start with 0IM or 0IW, which are the codes for self, followed by a sequence of three-digit codes that describe intermediate relationships from an individual to their kin. For instance, father is expressed as 0IM1AM or 0IW1AM, meaning one’s man parent. Paternal grandmother can be broken down into one’s father’s mother so is given codes 0IM1AM1AW or 0IW1AM1AW. Using this code offers an advantage of logically identifying interpersonal relationships, whereas a traditional code system, relationship to head of household, is unidirectional and even loses information on relationships between members of the household or inter-household relationships. Specifically, inverse relationship codes and extended relationship codes can be derived using this new family code system through intuitive formula (Figure 1). Moreover, it also allows the immediate calculation of degree of kinship as well as type of kinship such as direct ancestor, direct descendant, collateral blood relatives, spouse, and relatives by marriage.

Major variables of the family tree DB are shown in Table 4. As the family tree DB is based on inter-personal relationships, each individual can be either a subject or someone’s family member. Hence, to distinguish such relationships, each record specifies who is a subject (Subject ID), who is a family member (Family ID), and the relationship of the family member to the subject (Family code).

### Ethics statement

This dataset was drawn from a retrospective cohort based on administrative data, and separate patient recruitment procedures were not carried out. As the data were de-identified, the consent of the subject and direct contact were not applicable.

## DATA RESOURCE USE

**Figure 2.**
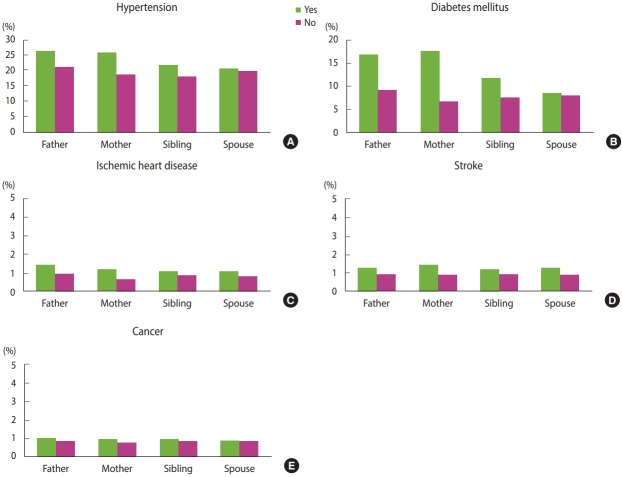
Disease prevalence rate in 2017 according to the disease (A) hypertension, (B) diabetes mellitus, (C) ischemic heart disease, (D) stroke, and (E) cancer history by family relationship in the family tree database.

Since the family tree DB was only recently established in 2018, and access to the DB is limited even for research purposes, very few studies have been conducted using this DB thus far. The family tree DB will be most effective when used with the NHID rather than used independently. An example of this is the systematic analysis of family history of disease shown in Figure 2. We analyze history of medical diagnoses and treatments from the medical claims data in conjunction with the family tree DB. We focus on prevalence rates of hypertension, diabetes, ischemic heart disease, cerebrovascular disease, and cancer by degree of kinship. Using the medical records by 2017, we define patients with each disease as follows: patients with hypertension and with diabetes are respectively defined as those who have disease codes (I10-I15, E10-E14) and prescription medicines for hypertension and diabetes, and patients with the other diseases as those who are admitted to a hospital with the disease codes for ischemic heart disease (I20-I25), cerebrovascular disease (I60-I69), cancer (C00-C97 for primary diagnosis only). The prevalence rates are standardized by five-year age groups of the entire population eligible for health insurance in 2015. For all the analyzed diseases, the prevalence rates are higher in the presence of family history, especially when the mother’s side has a history of the same disease.

## STRENGTHS AND WEAKNESSES

The family tree DB is a unique DB in Korea that contains family relations up to the fourth degree of kinship for the entire population using health insurance eligibility and resident register data. In addition, it allows more complete identification of family relationships between two individuals even if their relationship is not identified from the source data in which there are only relationships between head and a member of household. This new DB is expected to serve as a fundamental source to provide empirical evidence for solving various social problems through its application to medical research as well as social policies. The limitations of this project are as follows. Due to restrictions of available data (e.g. not being able to use the family relations register, etc.), family relations among the elderly or those who passed away before the source data were created cannot be clearly determined. In addition, due to limitations of the source data based on the head of household-members of household relationship, non-determinable codes may occur when extended relationship codes are created. Finally, as there is a lack of studies using this DB, additional comparison studies should be conducted to validate its accuracy.

## DATA ACCESSIBILITY

The family tree DB is part of the NHID and the general principles regarding access to the NHID are noted in the health insurance data share service information at http://nhiss.nhis.or.kr. When a researcher makes an online request for data based on a research proposal approved by institutional review board, the data are provided after approval by the data service review board. However, the family tree DB can be limitedly provided for studies with high public value in order to maximize personal information protection.

## Figures and Tables

**Table 1.** Parent-child and grandparent-child matching rates of the family tree by birth year of child

Birth year of child	Total (n)^1^	Parent			
		Matching rate (%)			
		Father	Mother	Any father or mother^2^	Any father or mother or grand-father or grand-mother^2^
Total	52,712,258	59.3	66.0	67.9	68.5
Men					
2010s	1,832,687	95.3	95.4	97.3	99.6
2000s	2,576,650	97.7	97.7	98.9	99.8
1990s	3,689,005	95.2	95.7	96.7	97.2
1980s	3,831,362	89.1	91.8	93.7	94.3
1970s	4,335,943	76.0	86.7	89.7	90.4
1960s	4,442,634	40.9	58.5	61.6	62.0
-1950s	5,682,554	14.0	31.7	34.2	34.3
Women					
2010s	1,737,751	95.2	95.4	97.3	99.6
2000s	2,393,192	97.5	97.6	98.8	99.8
1990s	3,279,741	95.8	96.5	97.5	98.1
1980s	3,549,234	89.3	92.7	94.5	95.1
1970s	4,169,971	69.0	78.8	81.5	82.2
1960s	4,367,663	19.8	28.8	30.5	30.7
-1950s	6,823,871	1.6	5.3	5.7	5.7

1 The population is all Korean residents who are eligible for health insurance in 2017.

2 Only those parents or grandparents who have been in the same household or in the same health insurance unit are counted.

**Table 2.** Family relationship characteristics of the Korean family tree database of the National Health Information Database in 2017

Birth year of subject	Total of subject (n)	Degree of kinship (family n)				Family type (family n)	
		1	2	3	4	Consanguinity	Affinity
Men							
2010s	1,832,687	3,525,580	3,797,925	812,624	137,143	8,228,839	44,433
2000s	2,576,650	5,123,533	6,436,856	1,800,865	583,624	13,765,732	179,163
1990s	3,689,005	7,294,082	8,021,793	2,295,048	948,343	18,176,279	446,777
1980s	3,831,362	9,411,122	7,493,191	1,963,043	623,295	18,034,669	2,974,384
1970s	4,335,943	13,808,062	9,686,874	2,898,052	263,247	23,242,758	6,702,472
1960s	4,442,634	12,864,887	5,139,510	1,819,799	75,599	17,592,909	6,384,494
-1950s	5,682,554	18,276,848	7,877,527	860,301	54,125	21,974,653	10,604,122
Women							
2010s	1,737,751	3,341,412	3,620,865	768,545	130,549	7,818,297	43,074
2000s	2,393,192	4,758,720	6,090,381	1,689,806	551,867	12,916,939	173,867
1990s	3,279,741	6,764,628	7,603,628	2,114,424	898,462	16,865,442	696,118
1980s	3,549,234	11,030,818	8,271,013	1,829,100	585,567	18,746,017	5,079,562
1970s	4,169,971	16,176,391	10,911,164	2,563,870	194,630	23,260,513	10,355,356
1960s	4,367,663	13,508,216	4,980,864	1,517,419	60,212	14,555,190	9,758,063
-1950s	6,823,871	22,991,039	13,485,353	1,171,750	65,998	27,375,520	15,839,506

**Table 3.** Three-digit basic family code for identifying interpersonal relationships in the family tree database

Code	Meaning
Panel A: first two digits	
0I	Self (zero degree)
0O	Spouse (zero degree)
1A	Parent (1st degree)
1D	Child (1st degree)
2A	Older sibling (2nd degree)
2D	Younger sibling (2nd degree)
2U	Sibling (2nd degree)
1a	Step parent (1st degree)
1d	Step child (1st degree)
Panel B: third digit	
M	Men
W	Women
X	Unspecified

**Table 4.** Variables and contents of the family tree database of the National Health Information Database

Variable	Variable name	Contents
TG_ID	Subject (target) ID	
FMLY_ID	Family ID	
TG_SEX_TYPE	Sex type of subject	
TG_BYEAR	Birth year of subject	
FMLY_SEX_TYPE	Sex type of family	
FMLY_BYEAR	Birth year of family	
FMLY_CD	Family code	Combination of 3-digit codes - more information in Figure 1
FMLY_DGR	Degree of kinship	0-4
FMLY_TYPE	Family type	C1: Close blood relatives
		C2: Non-close blood relatives
		A0: Spouse
		A1: Close relatives by affinity
		A2: Non-close relatives by affinity in law
		A3: Non-close relatives by affinity in old-law
		R1: Adoptive parents or children
HHRR_CD	History of same household	1: With same household and health insurance unit history
		2: With same household history only
		3: With same health insurance unit history only

## SUPPLEMENTARY MATERIALS

Korean version is available at http://www.e-epih.org/.
