# Supplementary material for: Family tree database of the National Health Information Database in Korea
Source: Epidemiol Health. 2019 Oct 1;41:e2019040. doi: 10.4178/epih.e2019040 (PMC6928464; doi:10.4178/epih.e2019040)
Supplement: Supplementary file 1 [file epih-41-e2019040-supplementary.pdf]

Volume: 41, Article ID: e2019040, 6 pages

https://doi.org/10.4178/epih.e2019040

Data profile: Family tree database of the National Health Information Database in Korea

Yeon-Yong Kim[1]*, Hae-young Hong[2]*, Kyu-Dong Cho[1], Jong Heon Park[3]

[1]Department of Big Data, National Health Insurance Service, Wonju, Korea; [2]Department of Economics, University of Wisconsin, Madison, WI, USA; [3]Department of Benefits Strategy, National Health Insurance Service, Wonju, Korea

* Equally contributed as a first author

Correspondence: Jong Heon Park

Department of Benefits Strategy, National Health Insurance Service, Wonju, Gangwon, Republic of Korea

E-mail: parkjh@nhis.or.kr

Running title: Family tree database of the NHID

Volume: 41, Article ID: e2019040, 6 pages

https://doi.org/10.4178/epih.e2019040

Abstract:

가족관계는 질병 가족력 등 생물학적 측면에서 중요한 요인으로 알려져 있으며 최근에는 저출산 및 고령화, 독거노인 및 1 인 가구 등 사회구조적 측면에서도 중요 요인으로 부각되고 있다. 가족관계도 DB 는 건강보험 가입자 자격자료와 주민등록자료를 이용하여 가족관계도를 논리적으로 표현한 가족관계 코드에 기반하여 2018 년도에 구축된 DB 다. 2017 년 기준 약 5,271 만 명 중 2010 년대에 출생한 대상자의 95% 이상에서 부모 및 조부모가 연계되어 있다. 대상자와 가족의 성별, 출생년도, 가족관계(3 자리 기본코드의 연속으로 역 관계 및 확장 관계 코드 생성 가능), 촌수(최대 4 촌) 등이 변수로 구축되어 있다. 가족력이 있는 대상에서의 환자 비율을 비교하면, 고혈압, 당뇨병, 허혈성 심질환, 뇌혈관질환, 암에서 모두 가까운 가족력이 있을 때 질환유병 비율이 높았다. 가족관계도 DB 는 과거 자료 축적의 한계로 일부 미연계 대상이 있을 수 있으며, 세대주-세대원 관계에 기반한 원천 자료의 한계로 일부 가족관계를 명확히 파악하기 어렵다. 가족관계도 DB 는 국민건강정보 DB 내에 구축되어 있으며, 연구자가 온라인 홈페이지(http://nhiss.nhis.or.kr)를 통해 자료를 신청하면 자료제공심의위원회를 거쳐 자료가 제공된다. 단, 가족관계도 DB 는 개인정보 보호측면에서의 안정성 극대화를 위해 공익적 활용가치가 높은 정책연구에 한해 가공된 형태로 제한적으로 제공될 예정이다.

Key words: family, database, family relations, interpersonal relations

Volume: 41, Article ID: e2019040, 6 pages

https://doi.org/10.4178/epih.e2019040

Introduction

한국의 건강보험(National Health Insurance)은 사회보험으로 1989 년부터 전국민을 대상으로 하고 있으며, 요양기관 당연지정제로 인하여 모든 공급자가 건강보험 대상에 포함된다. 국민건강보험공단[이하 공단, National Health Insurance Service (NHIS)]은 건강보험을 운영하는 단일 보험자(single insurer)이며, 주된 지불제도로는 행위별 수가제(fee-for-service)가 운영되고 있다[1].

공단은 전국민의 보험 자격 관리 및 보험료 부과를 위한 인구학적 정보(출생, 사망, 거주지, 세대구성 등), 사회경제학적 정보(직장, 소득, 재산 등), 장애등록내역 등의 정보와 함께 국가 건강검진제도(영유아, 성인일반검진, 암검진 등) 운영 및 관리 주체로서 다양한 건강위험요인 정보를 보유하고 있다. 또한, 의료기관에서 이루어진 5 천만 명 전체 인구집단의 상세한 의료이용 내역(행위, 약제, 치료재료) 도 있다. 다양한 연구목적 자료요청 수요를 충족하기 위하여 공단은 2012 년 주민등록번호 등 식별 정보가 제외된 연구용 DB 인 국민건강정보 DB 를 구축하였다[2]. 2018 년에는 인구사회학적 정보 등 다양한 변수에 대한 제공 수요 충족을 위하여 인구(가족관계 등), 지리, 사회, 경제, 사회적 자원, 건강행태, 의료이용의 형태로 국민건강정보 DB 를 재구축하였다.

가족관계는 질병 가족력 등 생물학적 측면에서 중요한 요인으로 알려져 있으며[3], 최근에는 저출산 및 고령화 등 사회구조적 측면에서도 중요 요인으로 부각되고 있다. 독거노인 및 1 인 가구 등 가족구조와 관련된 건강 문제는 사회적 이슈로도 주목 받고 있다[4]. 또한 사회적 네트워크(social network) 등 사회적 신뢰 측면에서 가족관계는 정신건강에도 영향을 미치는 것으로 보고되고 있다[5]. 학술연구 외에 정책기반 근거 연구에서도, 효과적인 건강증진 및 사회정책 수립을 위하여 정확한 가족관계도에 기반한 접근은 필수적이다.

하지만 가족관계도를 정확히 파악할 수 있는 자료원이 충분하지 않다는 한계점이 존재하였다. 기존 설문조사 기반 자료원 중에도 가족관계를 파악하고 있는 자료가 있지만 대부분 세대주와의 관계(relationship to head of household)만을 수집하기 때문에 세대주-세대원 이외의 관계를 파악하는 데 한계가 있다. 공단이 보유하고 있는 건강보험 가입자 자격자료와 주민등록자료도 세대주와의 관계 코드만 존재하는 유사한 한계점이 존재한다. 이를 극복하고자 가족관계도를 논리적으로 표현할 수 있는 코드를 개발하여 '개인 간 상호관계(inter-personal relationship)' 형태로 전국민의 가족관계에 대한 DB 를 구축하였다.

Volume: 41, Article ID: e2019040, 6 pages

https://doi.org/10.4178/epih.e2019040

Data resource area and population coverage

가족관계도 DB(family tree DB)에서는 국민건강정보 DB 내의 건강보험 자격자료와 행정전산망 자료를 비교·상호보완하여 정확도 높은 관계도를 도출하였다. 국민건강정보 DB 가 전체 국민을 대상으로 하고 있어 가족관계도 DB 도 동일하게 전국민을 대상으로 하고 있으나 보유 자료원의 시기적 한계로 인하여 2002 년 이전의 가입자 정보와 2004 년 이전의 행정전산망 정보는 일부 누락되어있다. 가족관계도 DB 는 가계도 형태의 DB 이므로 기준년도가 별도로 존재하지 않는다. 그러나, 연도별 자격자료와 연계하면 특정 연도의 가족관계 빈도를 파악할 수 있다. 예를 들어, 가족관계도 DB 에는 A 가 B 의 아버지라는 관계만 존재하지만, 2015 년 자격자료와 연계하면 해당 년도에 몇 개의 부-자 관계가 존재하는지 파악할 수 있다. Table 1 에서는 2017 년 기준 인구의 가족관계도 DB 의 출생연도별 부모 또는 조부모 연계 비율을 제시하였다. 2010 년대 출생자는 99.6%에서 부모 또는 조부모가 연계되나, 1950 년대 이전 출생자는 남성 34.3%, 여성 5.7%에서 부모 또는 조부모가 연계되는 것을 확인할 수 있다.

가족의 종류에 따른 2017 년 기준 대상자 빈도는 Table 2 에 제시되어 있다. 가족관계도 DB 는 4 촌까지의 관계를 파악할 수 있으며, 1 촌 관계는 남녀 모두 1950 년 이전 출생자 중에서, 2 촌 관계는 남성은 1970 년대 출생자, 여성은 1950 년 이전 출생자 중에서, 3 촌 관계는 남녀 모두 1970 년대 출생자 중에서, 4 촌 관계는 남녀 모두 1990 년대 출생자 중에서 가장 많이 파악되는 것으로 나타났다. 혈족(consanguinity)은 남성 1970 년대 생, 여성은 1950 년대 이전 출생자 중에서, 인척(affinity)은 남녀 모두 1950 년대 이전 출생자에서 가장 많이 파악되는 것으로 나타났다. 즉, 전반적으로는 고령자일수록 파악되는 가족 대상 인원이 많다는 것을 의미한다.

Measures

Family code and variables

세대주와의 관계로부터 개인간 친족관계로 확장을 하기 위하여 새로운 친족코드(family code) 체계를 고안하였다. 친족코드는 본인으로부터 상대방에 도달하기까지 가장 최소한으로 필요한 연결 관계(Linking kins)를 3 자리 기본코드(3-digit atomic code)들의 연속으로 나타낸 것이다. 3 자리 코드의 첫째 자리는 촌수를 의미하며, 두 번째 자리는 본인/배우자/연장자/연소자 등을 구분하고, 세 번째 자리는 성별을 나타낸다 (Table 3). 모든 친족코드는 본인 코드인 0IM(W)로 시작하고, 상대방까지 필요한 기본 관계들이 뒤따라 이어지는 형태이다. 예를 들어, '아버지'는 본인의 부이기 때문에

https://doi.org/10.4178/epih.e2019040

‘0IM1AM’ 혹은 ‘0IW1AM’으로 코드가 부여된다. ‘조모’는 본인의 부의 어머니로 분해되기 때문에 ‘0IM1AM1AW’ 혹은 ‘0IW1AM1AW’로 코드를 부여한다. 이 코드를 사용하면 기존 세대주와의 관계에서 단 방향으로 구축된 관계코드와 달리 개인과 개인 간 관계를 논리적으로 명확화할 수 있는 장점이 있다. 구체적으로, 역 관계 및 확장 관계 코드는 이 코드로부터 공식에 의해 도출될 수 있다(Figure 1). 또한 코드로부터 곧바로 촌수 계산과 직계존속, 직계비속, 방계혈족, 배우자, 인척 등의 친족 분류가 가능하다.

가족관계도 DB 의 주요 변수는 Table 4 에 제시되어 있다. 가족관계도 DB 는 개인 간 상호관계에 기반한 DB 이므로 전국민 개개인이 대상자가 될 수도, 누군가의 가족이 될 수도 있다. 따라서 이 관계를 구분하기 위하여 대상자 기준으로 가족관계 및 가족 ID 를 명시하였다.

Data Resource use

가족관계도 DB 는 2018 년 구축되었고, 연구용 목적으로도 제한적인 공개가 이루어지고 있어 이를 직접 활용한 연구 사례는 거의 없다. 다만 가족관계도 DB 는 독립적으로서가 아닌 건강보험 빅데이터인 국민건강정보 DB 와 연계되었을 때 파급효과가 극대화되며, 질병 가족력에 대해 탐색적으로 분석한 결과를 Figure 2Figure 로 제시하였다. 진료내역에서의 상병코드 및 약제 청구내역과 가족관계도 DB 를 연계하여 분석하였으며, 고혈압, 당뇨병, 허혈성심질환, 뇌혈관질환, 암에 대하여 그 대상자의 가족력 유무에 따른 2017 년도 기준 질환 유병 비율을 분석하였다. 국민건강정보 DB 내 2017 년도 이전까지의 이력을 바탕으로 고혈압과 당뇨병은 상병코드(I10-I15, E10-14)와 약제처방이 있었던 경우를, 허혈성심질환(I20-25), 뇌혈관질환(I60-69)은 상병코드로 입원한 경우를, 암의 경우는 주상병(C00-C97)으로 입원한 경우 중 산정특례 등록환자로 정의하였다. 유병 비율은 연령에 대하여 5 세 단위로 직접 표준화를 수행하였으며, 표준인구는 2015 년 건강보험 적용인구 전체로 설정하였다. 분석한 모든 질환에서 가족력이 있는 경우 없는 경우보다 질환의 비율이 높았으며, 특히 배우자보다는 부 또는 모에서 가족력이 있을 때 질환 비율이 더 높은 것으로 나타났다.

Strengths and weaknesses

가족관계도 DB 는 전국민의 4 촌 이내 가족관계를 건강보험 가입자정보와 행정전산망 자료를 통하여 재구축한 국내 유일한 자료다. 또한 세대주와의 관계코드로 구성되었던 기존의 가족관계를 논리적 체계를 갖춘 개인 간 상호관계 형태로 전환하여 명확한

Volume: 41, Article ID: e2019040, 6 pages

https://doi.org/10.4178/epih.e2019040

가족과 가족과의 관계 파악이 가능하게 되었다. 이는 의학적 연구에의 활용은 물론, 사회정책적 활용을 통한 다양한 사회문제 해결의 기초가 될 근거자료의 기반이 된다. 한계점으로는 가족관계 등록부를 사용하지 못하는 등 가용 자료의 한계로 인하여 고령층 또는 자료구축 기간이전에 사망한 가족 관계가 명확하게 파악되기 어려운 점이 있다. 또한 세대주-세대원 관계로 주어진 원천 자료의 한계로 확장 관계 코드를 생성할 때 비확정적인 코드가 발생할 수 있다. 또한 이 DB 를 활용한 연구가 아직은 부족하므로, 정확성을 검증하기 위한 다양한 비교 연구는 추가적으로 이루어져야 할 필요가 있다.

Data Accessibility

가족관계도 DB 는 국민건강정보 DB 내에 구축되어 있으며 국민건강정보 DB 의 일반적 제공원칙은 건강보험 자료공유서비스(http://nhiss.nhis.or.kr) 에 제시되어 있다. IRB 심의를 받은 연구계획서를 바탕으로 연구자가 온라인으로 자료를 신청하면 자료제공심의위원회를 거쳐 자료가 제공된다. 다만 가족관계도 DB 는 개인정보 보호측면에서의 안정성을 극대화 할 필요가 있어 맞춤형 연구 중에서도 공익적 활용가치가 높은 정책연구에 한해 가공된 형태로 제한적으로 제공될 예정이다.

References

1. Kwon S. Thirty years of national health insurance in South Korea: lessons for achieving universal health care coverage. Health Policy Plan 2009;24:63-71.

2. Seong SC, Kim YY, Khang YH, Park JH, Kang HJ, Lee H, et al. Data resource profile: the national health information database of the National Health Insurance Service in South Korea. Int J Epidemiol 2017;46:799-800.

3. Hunt SC, Gwinn M, Adams TD. Family history assessment: strategies for prevention of cardiovascular disease. Am J Prev Med 2003;24:136-142.

4. Hughes ME, Waite LJ. Health in household context: living arrangements and health in late middle age. J Health Soc Behav 2002;43:1-21.

5. Fiori KL, Antonucci TC, Cortina KS. Social network typologies and mental health among older adults. J Gerontol B Psychol Sci Soc Sci 2006;61:P25-P32.

Volume: 41, Article ID: e2019040, 6 pages

https://doi.org/10.4178/epih.e2019040

Figure legends

Figure 1. Examples of family code for the inverse and extended relationship

(A) **An example of deriving the family code for the inverse relationship**

(B) An example of deriving the family code for an extended relationship

*** 3-digit atomic codes (ex. 0IM, 1AW) are given in three digits according to the family extension rules and the rules are specified in Table 3.**

Figure 2. Disease prevalence rate in 2017 according to the disease history of family relationship in the family tree DB

(A) **Hypertension (B) Diabetes mellitus (C) Ischemic heart disease (D) Stroke (E) Cancer**
